# Thioredoxin reductase-1 facilitates cellular plasticity and stemness mediated by TGF-β1 in non-small cell lung cancer

**DOI:** 10.1080/15384047.2026.2724630

**Published:** 2026-09-08

**Authors:** Yaw-Dong Lang, Shin-Yuan Gu, Jou-Ho Shih, Chun-Jung Lin, Hua-Hsin Chiang, Tsai-Yu Lin, Yu-Ching Lee, Keng-Chang Tsai, Jui-Tin Chang, Cheng-Ying Chu, Yuh-Shan Jou, Teh-Ying Chou

**Affiliations:** a Division of Basic Chinese Medicine, National Research Institute of Chinese Medicine, Ministry of Health and Welfare, Taipei, Taiwan; b Graduate Institute of Clinical Medicine, College of Medicine, Taipei Medical University, Taipei, Taiwan; c Department of Medical Research, Taipei Medical University Hospital, Taipei, Taiwan; d Precision Medicine Research Center, Center for Precision Health and Quantitative Sciences, Taipei Medical University Hospital, Taipei, Taiwan; e Department of Genetics, Stanford University School of Medicine, CA, USA; f Ph.D. Program for Cancer Molecular Biology and Drug Discovery, College of Medical Science and Technology, Taipei Medical University, Taipei, Taiwan; g TMU Research Center of Cancer Translational Medicine, Taipei Medical University, Taipei, Taiwan; h PhD Program in Medical Biotechnology, College of Medical Science and Technology, Taipei Medical University, Taipei, Taiwan; i School of Medicine, Fu Jen Catholic University, New Taipei City, Taiwan; j Department of Internal Medicine, Nutrition Department, Division of Nephrology, Shin Kong Wu Ho-Su Memorial Hospital, Taipei, Taiwan; k CRISPR Gene Targeting Core, Taipei Medical University, Taipei, Taiwan; l Institute of Biomedical Sciences, Academia Sinica, Taipei, Taiwan; m Department of Pathology, School of Medicine, College of Medicine, Taipei Medical University, Taipei, Taiwan; n Department of Pathology and Laboratory Medicine, Shin Kong Wu Ho-Su Memorial Hospital, Taipei, Taiwan

**Keywords:** Non-small cell lung cancer (NSCLC), thioredoxin reductase 1 (TXNRD1), cellular plasticity, epithelial–mesenchymal transition (EMT), transforming growth factor beta 1 (TGF-β1), cancer stemness, tumor metastasis

## Abstract

**Background:**

Cellular plasticity and epithelial–mesenchymal transition (EMT) promote the initiation and progression of non-small cell lung cancer (NSCLC). Thioredoxin reductase 1 (TXNRD1), a key redox enzyme, has been linked to malignancy, but its mechanism in NSCLC remains unclear. We examined whether TXNRD1 regulates TGF-β1 autocrine signaling to drive EMT and stemness.

**Materials:**

Stage-progression gene profiles were analyzed in the TCGA and GEO databases with an emphasis on redox gene families. TXNRD1 was manipulated by overexpression or knockdown in A549, H226, and H1299 cells, followed by migration/invasion, spheroid assays, ELISA for cytokines, and RT-qPCR/Western blot for EMT markers. RNA-seq with pathway enrichment analyses was used to identify downstream programs. An orthotopic lung cancer mouse model was established using TXNRD1-WT cells, TXNRD1-deficient cells, and TXNRD1-deficient cells treated with TRi-1. Tumor progression was monitored by bioluminescence imaging at 6 and 12 weeks after transplantation.

**Results:**

TXNRD1 expression was ~2-fold higher in advanced-stage NSCLC and was validated in tumor tissues. CRISPR/Cas9 or siRNA knockdown reduced EMT-associated genes and decreased TGF-β1 production in A549 and H226 cells. TXNRD1 overexpression increased EMT and stemness markers and produced larger, more compact spheres with higher sphere numbers in H1299 cells. RNA-seq indicated the TXNRD1 pathway activates the TGF-β1 pathway to promote EMT, motility, and stemness via an autocrine loop; knockdown or TXNRD1 inhibition suppressed metastatic tumor growth in vivo.

**Conclusions:**

Our study identifies TXNRD1 as a crucial regulator of cellular plasticity and metastasis in NSCLC via the TGF-β1 pathway, suggesting that targeting TXNRD1 may reduce metastatic potential and improve patient survival.

## Introduction

Lung cancer is the most common malignancy worldwide, with an estimated 2 million cases and 1.7 million deaths annually, making it the leading cause of cancer-related mortality.^1^ Pathologically, approximately 80–85% of lung cancers are classified as non-small cell lung cancer (NSCLC), which primarily includes lung adenocarcinoma and lung squamous cell carcinoma.^2^ Over the past few decades, lung adenocarcinoma, or lung squamous cell carcinoma, has emerged as the predominant histological subtype of NSCLC, particularly in women and never-smokers.^3^ The tumorigenesis of NSCLC is multifactorial and influenced by cigarette smoking, second-hand smoke exposure, environmental factors, and inherited genetic susceptibility.^4^

NSCLC represents the major subtype of lung cancer for which extensive genomic and clinical datasets are publicly available, enabling integrated genomic analyses.^5^ Although previous efforts have focused on identifying mutated coding genes for targeted therapies, many bioinformatics studies have lacked mechanistic and experimental validations, limiting their translational relevance.^6^ ^,^ ^7^ Although patients are enrolled in genome-driven clinical trials, only a minority of patients with advanced NSCLC benefit from current targeted therapies. Therefore, comprehensive identification of prognosis-associated driver genes through functional prediction and mechanistic validation is essential for the development of effective anticancer strategies.

Cancer cells frequently exhibit elevated oxidative stress due to a dysregulated metabolism and increased proliferative demand.^8^ ^,^ ^9^ These cells adapt to increasing levels of reactive oxygen species (ROS) by upregulating antioxidant molecules, limiting ROS levels to the threshold of cytotoxicity.^10^ Paradoxically, excessive antioxidant supplementation promotes tumor growth, underscoring the complexity of redox regulation in cancer.^11^ Intracellular oxidative stress can be regulated by the activation of the thioredoxin (TXN) and glutathione (GSH) systems.^12^ The thioredoxin system comprises TXN, thioredoxin reductases (TXNRD1, TXNRD2, and TXNRD3), thioredoxin-interacting protein (TXNIP), and NADPH and plays a central role in maintaining redox homeostasis.^13^

TXNRD1, a cytosolic flavin adenine dinucleotide oxidoreductase, is a selenoprotein that performs diverse antioxidant and redox regulatory functions.^14^ It is frequently overexpressed in multiple cancer types and has been proposed as a critical driver of tumor cell proliferation and survival.^15^ ^,^ ^16^ In lung cancer, TXNRD1 levels are correlated with NRF2 pathway activation and tumor recurrence, highlighting its role in disease progression.^17^ Furthermore, inhibition of TXNRD1 by small interfering RNA (siRNA) library screening sensitized NSCLC cells to Keap1-dependent lethality induced by Akt kinase.^18^ USF2 is a transcriptional suppressor of TXNRD1 that directly interacts with the TXNRD1 promoter to activate Akt/mTOR signaling in hepatocellular carcinoma.^19^ Pharmacological inhibition of TXNRD1 using TRi-1 induces ROS accumulation and effectively suppresses tumor growth in liver cancer.^20^ ^,^ ^21^ Evidence indicates that ROS play a critical role in epithelial–mesenchymal transition (EMT) engagement, signaling, and tumor progression.^22^ Notably, TXN and TXNRD1 are crucial mediators of the TGF-β1/Akt/GSK-3 axis, promoting EMT in salivary adenoid cystic carcinoma.^23^ However, the role of TXNRD1 in NSCLC progression remains largely unexplored. This study aimed to elucidate the functional role of TXNRD1 in cellular plasticity after tumor progression and uncover the underlying molecular mechanisms. Using The Cancer Genome Atlas (TCGA) and Gene Expression Omnibus (GEO) datasets, we systematically analyzed redox system-related genes, including those in the thioredoxin, superoxide dismutase, and glutathione reductase families. Among these genes, TXNRD1 emerged as the top-ranked candidate, exhibiting a two-fold increase in expression in stage IV compared with stage I NSCLC. Elevated TXNRD1 expression was confirmed in advanced-stage NSCLC tissues using a human tissue array.

To investigate the functional significance of TXNRD1, we explicitly state that TXNRD1 has been linked to general malignancy; its role in regulating TGF-β1 autocrine signaling to drive EMT and stemness in NSCLC was previously unknown.

Regarding the rationale for using multiple cell lines, we have added a clarifying paragraph explaining the complementary design: A549 cells were chosen for CRISPR/Cas9-mediated loss-of-function studies owing to their high endogenous TXNRD1 expression, whereas H1299 cells were used for gain-of-function overexpression experiments because of their low basal TXNRD1 levels. Additionally, H226 cells (squamous cell carcinoma) were included to extend our findings beyond adenocarcinoma and validate generalizability across NSCLC subtypes, while H1975 cells were employed to examine TGF-β1-induced transcriptional changes in the context of TXNRD1 modulation. This complementary strategy strengthens the mechanistic conclusions across histological subtypes. *TXNRD1* knockdown decreased TGF-β1 levels, as determined by a growth factor array and enzyme-linked immunosorbent assay (ELISA). We further validated downstream TGF-β1 signaling components by RT-qPCR following TXNRD1 overexpression in H1975 cells and TXNRD1 knockdown in A549 cells. TGF-β1-induced EMT models were used to assess transcriptional changes in EMT-related genes, and RNA sequencing revealed that TXNRD1 regulates the TGF-β1/SMAD signaling pathways. Finally, using an orthotopic lung cancer mouse model, we demonstrated that TXNRD1 knockdown suppressed tumor growth and metastasis, highlighting TXNRD1 as a potential therapeutic target for limiting EMT-driven tumor progression in NSCLC. These results suggest that targeting TXNRD1 may offer a promising therapeutic strategy to prevent or treat tumor progression following EMT, paving the way for potential clinical applications in NSCLC.

## Results

### TXNRD1 is the most significant stage progression-related gene from patients with NSCLC

**Figure 1.**
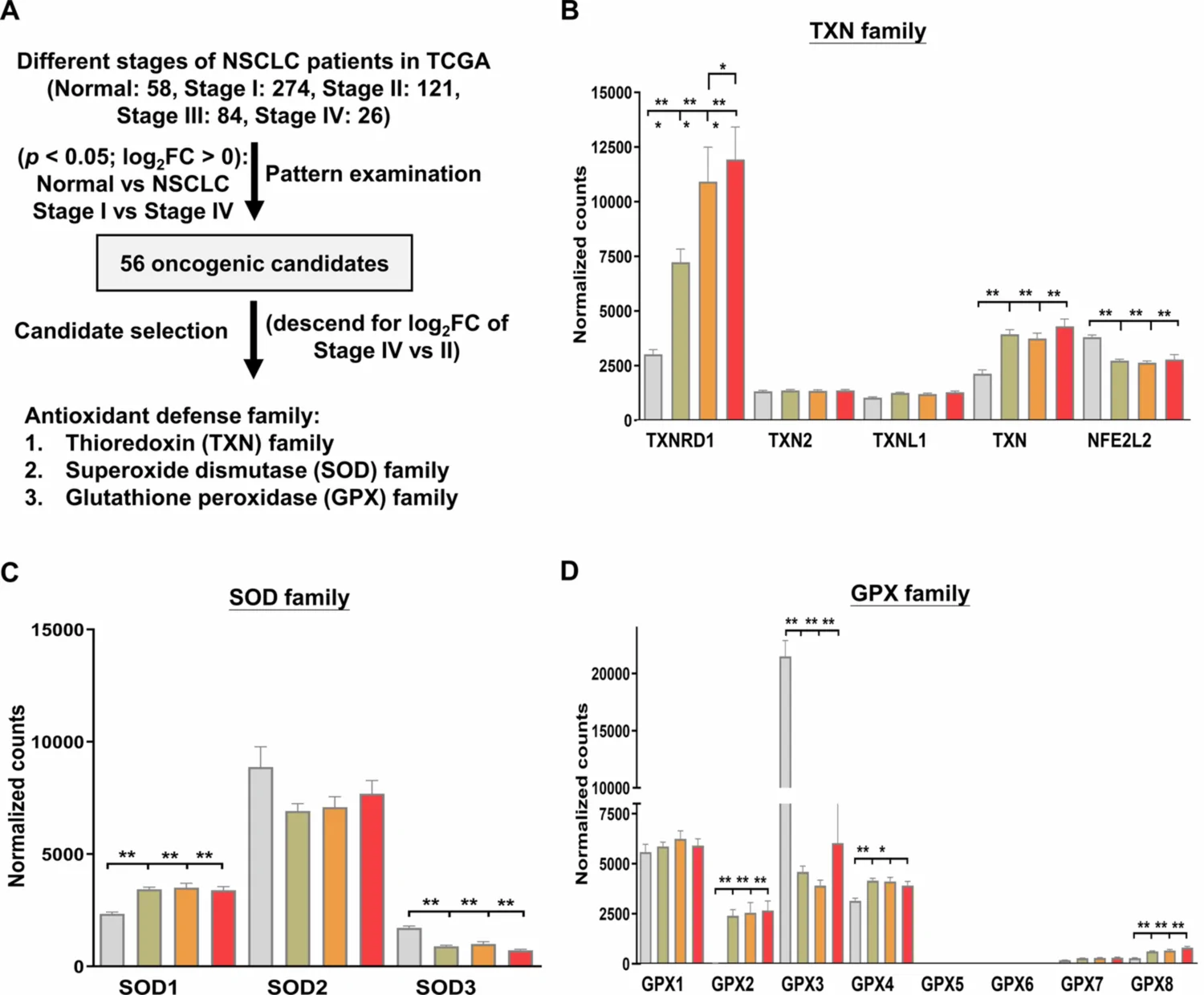
TXNRD1 is the most significant stage progression-related gene from NSCLC patients. (A) The cohort description of each patient-matched sample, including lymph-node metastases, primary NSCLC tumors, and adjacent normal lungs, was analyzed. The bulk RNA-seq data were collected to profile transcript abundances from the TCGA public database (https://ucsc-xena.gitbook.io/project/public-data-we-host/tcga flowchart for TXNRD1 selection from NSCLC patients in the TCGA cohort. According to the criteria of comparing normal samples with cancer samples and comparing stage I with stage IV, using log2 fold change > 0 and *p* < 0.05, we identified 56 overlapping DEGs as oncogenic candidates. To further prioritize the candidates from oncogenic candidates, the order of stage IV is reversed compared to stage II. Among these oncogenic candidates, we further focused on the antioxidant defense system family in the redox system, including the thioredoxin (TXN) family, superoxide dismutase (SOD) family, and glutathione peroxidase (GPX) family. Pattern selection for 56 oncogenic candidates by the *p*-value of the comparison between different groups. (B–D) The number of normalized counts showed that TXNRD1 is most upregulated in metastasis stages of NSCLC patients (red bar: stage III and IV) versus their primary tumor (brown and yellow bar: stage I and II) and adjacent normal stage (grey bar) compared to TXN family (B), SOD family (C), and GPX family (D) from TCGA public datasets. All data statistics were based on *p < 0.05, **p < 0.01, and ***p < 0.001 based on the ANOVA multiple comparison test.

To identify oncogenic candidates associated with disease progression in NSCLC, we analyzed transcriptomic datasets from TCGA at different clinical stages, as illustrated in Figure 1A. Candidate selection focused on antioxidant defense–related genes, including TXN, SOD, and GPX. Differential expression analysis identified numerous stage-dependent genes, with 370 genes upregulated in stage I, 711 in stage II, 356 in stage III, and 56 in stage IV tumors compared with normal controls (data not shown). Among these candidates, TXNRD1 expression was significantly elevated in tumor tissues relative to normal controls in a stage-dependent manner (Figure 1B). In contrast, TXN2, TXNL1, TXN1, and NFE2L2 showed no consistent stage-associated changes. Within the SOD family, SOD1 expression was moderately elevated across tumor stages, reaching statistical significance in multiple comparisons, whereas SOD2 and SOD3 levels were reduced (Figure 1C). GPX2 expression was markedly increased in early-stage tumors (Figure 1D), and the expression of GPX4 and GPX8 was also upregulated. Collectively, these findings indicate heterogeneous expression patterns of redox-related genes during NSCLC progression, with TXNRD1 emerging as a prominent candidate associated with disease progression. TXNRD1 was selected for further investigation because it exhibited the most robust and consistent stage-dependent upregulation, suggesting a strong association with tumor progression. Given its established role in redox homeostasis, treatment resistance, and malignant transformation, TXNRD1 represents a plausible and clinically relevant oncogenic factor in NSCLC. To determine whether TXNRD1 correlates with advanced disease stage and poor survival, we performed multivariable Cox regression analyses adjusted for TNM stage, age, and sex. TXNRD1 expression remained significantly associated with overall survival in both the TCGA cohort (hazard ratio [HR] = 1.10 per log_2_ unit, 95% CI 1.02–1.19, *p* = 0.016) and the microarray cohort (HR = 1.12 per log_2_ unit, 95% CI 1.05–1.20, *p* = 0.001), confirming that TXNRD1 is an independent prognostic factor beyond standard clinical covariates (Table 1 and Supplementary Table 2).

**Table 1.** Multivariable Cox regression of overall survival in TCGA and microarray cohorts.

	TCGA_result (*N* = 482)			Microarray_Result (*N* = 801)		
Factor	Hazard Ratio	95% CI	*p-value*	Hazard Ratio	95% CI	*p-value*
TXNRD1 (log2)	1.102*	1.018–1.192	0.016	1.12*	1.046–1.199	0.001
Age	1.065	0.936–1.211	0.338	1.26	1.162–1.365	<0.001
Sex (Male vs. Female)	0.92	0.721–1.174	0.502	1.254	1.065–1.478	0.007
Stage II vs. Stage I	1.582	1.200–2.086	0.001	1.439	1.212–1.709	<0.001
Stage III vs. Stage I	2.32	1.662–3.239	<0.001	2.229	1.573–3.159	<0.001
Stage IV vs. Stage I	2.255	1.460–3.482	<0.001	2.425	1.518–3.873	<0.001

Multivariable Cox models were independently applied to the TCGA cohort (*N* = 482) and the combined microarray cohort (*N* = 801) to assess the relationship between TXNRD1 expression and overall survival. TXNRD1 expression was analyzed as a continuous variable on the log2 scale. The models were modified for age, gender (male vs. female), and pathological stage (Stage II-IV, with Stage I serving as the reference group). The findings are presented as hazard ratios (HRs), 95% confidence intervals (CIs), and related *p*-values. The asterisk represents the TXNRD1 HR calculated from a multivariable model that was adjusted for stage, age, and gender.

^*^ *Refers to the hazard ratio (HR) for TXNRD1 from the multivariable Cox model adjusted for stage, age, and sex.*

### TXNRD1 is associated with tumor recurrence in patients with NSCLC

**Figure 2.**
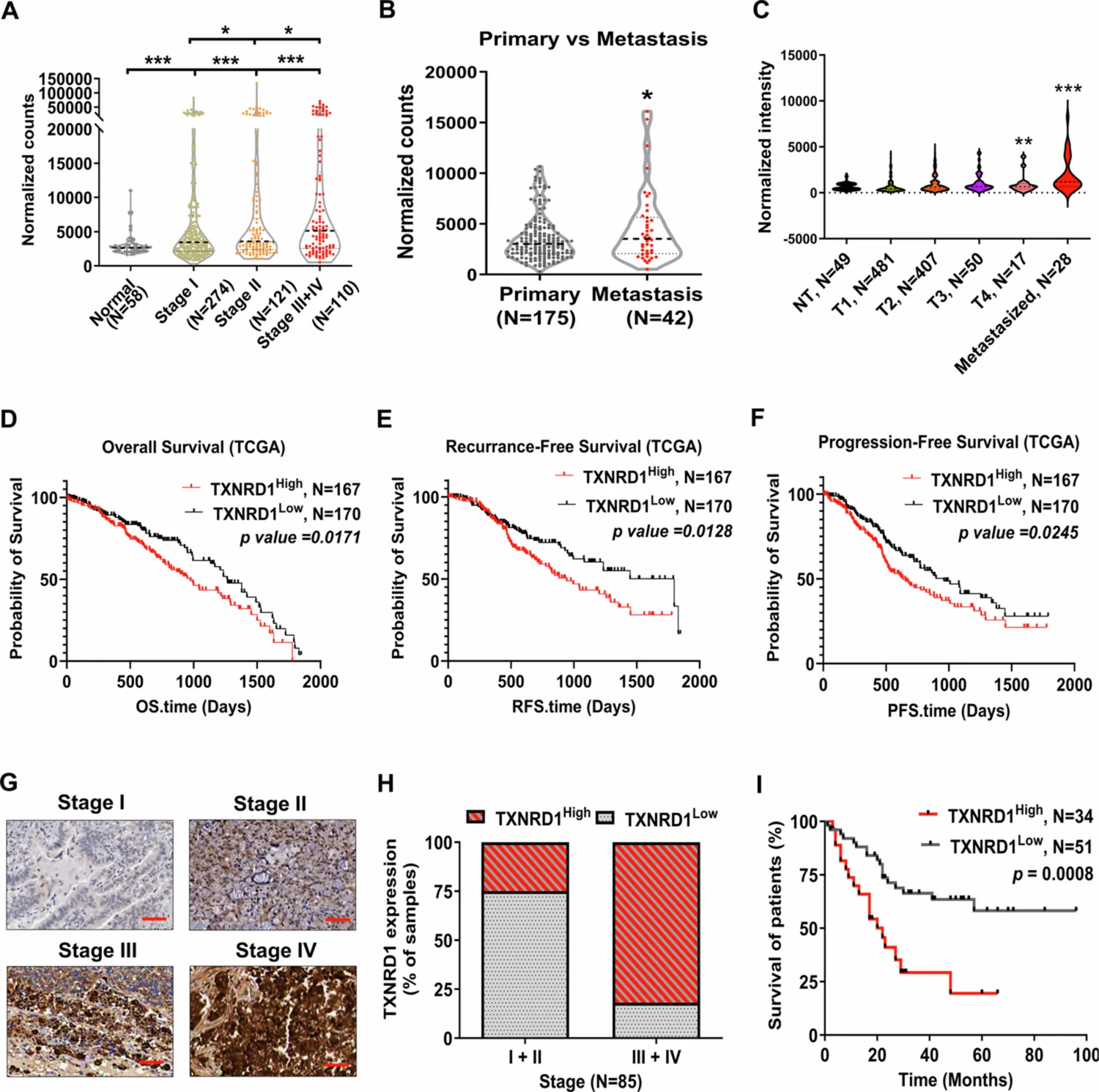
TXNRD1 levels are associated with tumor recurrence in NSCLC patients. (A) The number of normalized counts showed that expression profiles of TXNRD1 (red) in the metastasis stages of NSCLC patients versus their primary tumor and adjacent normal stage (grey) from the TCGA datasets. The lines in the center of the dots indicate the mean. (B) Normalized TXNRD1 transcript counts were compared between primary NSCLC (*N* = 175) and metastatic lesions (*N* = 42) from TCGA datasets. Violin plots display the distribution and density of expression values. The median and interquartile range are indicated. A statistically significant increase in TXNRD1 expression was observed in metastatic samples compared with primary tumors. (C) A total of 10 datasets of NSCLC T-stage samples were all downloaded from the GEO dataset (GSE14108, GSE14814, GSE27716, GSE30219, GSE31210, GSE33532, GSE31546, GSE37745, GSE43580, and GSE68465), and provided T-stage (primary tumor) information. The TXNRD1 normalized expression between different T-stage primary tumor samples and metastasized distant organ samples. All data statistics were based on *p < 0.05, **p < 0.01, and ***p < 0.001 based on the ANOVA multiple comparison test. (D–F) High versus low TXNRD1 expression profile was associated with poorer overall (D), recurrence-free (E), and progression-free (F) patient survival based on Kaplan–Meier analyses of NSCLC samples from TCGA datasets. The *N* values indicate the number of patients. (G) Representative IHC staining images of TXNRD1 expression from stage I to stage IV in human NSCLC tissues. The scale bar represents 50 μm. (H) Expression percentage of TXNRD1 (red color for higher expression) staining in early (I and II) and late-stage (III and IV) tumors in human NSCLC tissues based on IHC analysis. The statistical significance was calculated by the ANOVA multiple comparison test; *n* indicates the number of NSCLC patients. (I) Kaplan–Meier plot analysis of the overall survival of NSCLC patients based on low or high expression levels of TXNRD1.

Comprehensive analyses utilizing data from The Cancer Genome Atlas (TCGA) datasets revealed that the TXNRD1 expression level was substantially higher in NSCLC tumors than in normal tissues (Figure 2A). Expression was higher in metastatic samples than in primary tumors (Figure 2B). Independent validation using the GEO dataset (GSE14108) confirmed the sustained overexpression of TXNRD1 in NSCLC tumors (Figure 2C). In the TCGA cohorts, higher TXNRD1 expression was associated with lower overall survival (OS) (Figure 2D), shorter recurrence-free survival (Figure 2E), and poorer progression-free survival (Figure 2F). IHC analyses of commercial human NSCLC tissue arrays revealed a stage-dependent increase in TXNRD1 protein expression (Figure 2G). Quantitative analysis showed a higher proportion of TXNRD1-high tumors in advanced stages (III–IV) compared to early stages (I–II) (Figure 2H). Kaplan–Meier analysis of the IHC cohort revealed that patients with TXNRD1-high tumors had significantly lower survival rates than those with TXNRD1-low tumors (*p* = 0.0008, Figure 2I). Overall, these results suggested that increased TXNRD1 expression may be associated with tumor progression in NSCLC.

### TXNRD1 potentiates cellular motility and cancer stemness in NSCLC cells

**Figure 3.**
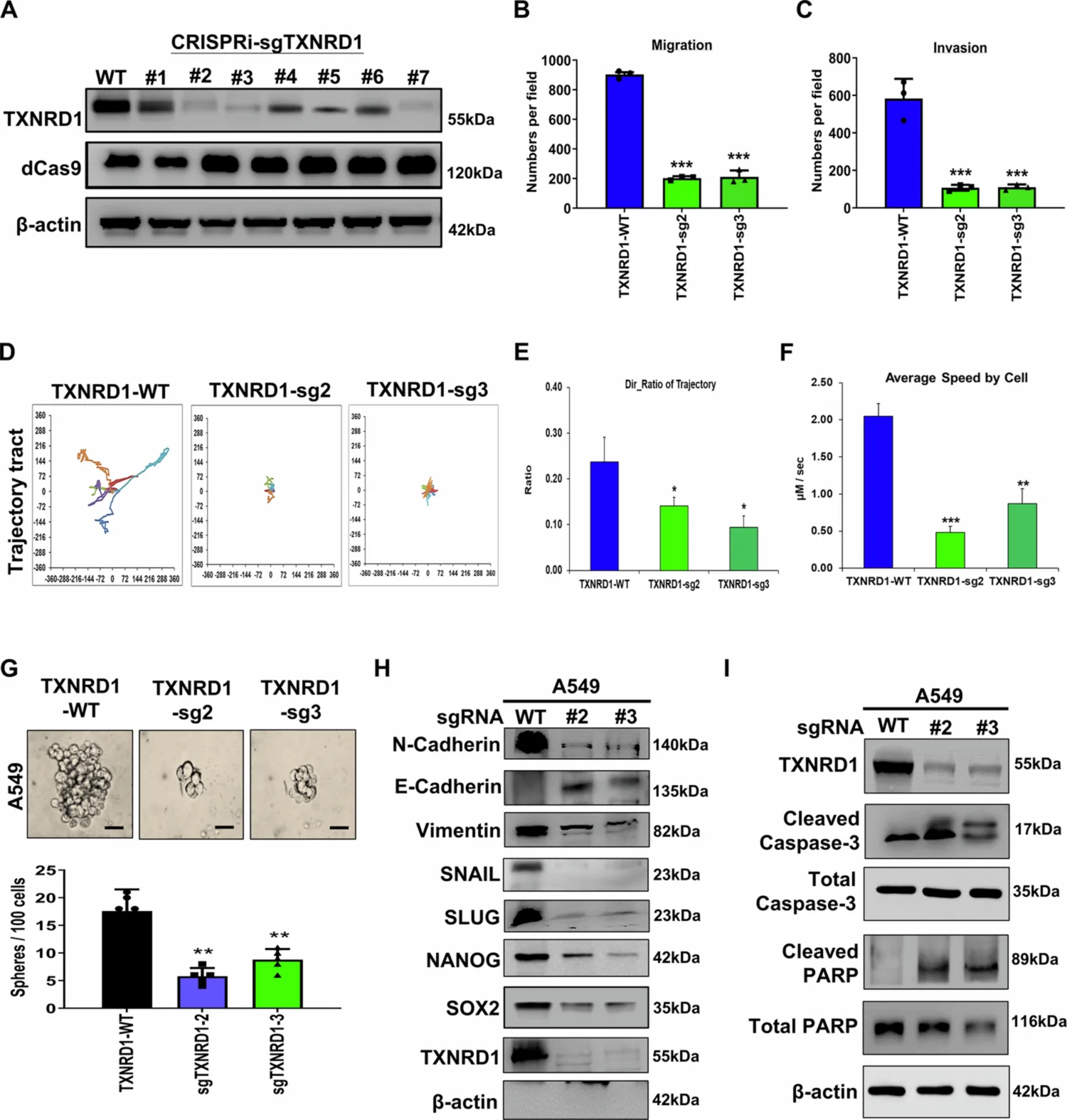
TXNRD1 potentiates cellular motility and cancer stemness in NSCLC cells. (A) CRISPR/Cas9 sgRNA targeting two sites in the TXNRD1 gene was delivered into A549 cells. Cell sorting of dCas9-GFP-positive cells from flow cytometry of TXNRD1-transfected A549 cells was required to create a single-cell clone. An immunoblot analysis was further performed to validate CRISPRi-TXNRD1 in A549 cells. Western blotting analysis in A549 cells to examine the protein expression of TXNRD1, Cas9, and internal control (*β*-actin). (B and C) Knockdown of TXNRD1 by CRISPR/ Cas9 decreased cell migration (B) and invasion (C) in A549 cells. (D–F) Time-lapse analysis of migratory ability by CRISPR knockdown of TXNRD1 in A549 cells, and the culture was traced for 16 h. The indicated group of cells was grown in a culture dish with Matrigel/Collagen coating. The representative wind rose plots of fields per well were taken at 3-minute intervals during 16 h, and the trajectory tracks of the indicated groups covered by A549 cells were plotted with the initial position of each trajectory track during the 16 h of recording. The directional ratios of trajectory per cell (E) and average cell velocity (F) were calculated from time-lapse video microscopy as described in the Materials and Methods. Data represent the mean ± SD (*n* = 5). All data statistics were based on *p < 0.05, **p < 0.01, and ***p < 0.001 calculated by one-way ANOVA with the Brown–Forsythe test. (G) Tumorspheres formation assay in knockdown of TXNRD1 by CRISPR/Cas9 in A549, including phase images (top) and graphic quantifications (bottom). Data are represented as mean ± SEM (*n* = 5). (H) Western blotting analysis of EMT and cancer stemness markers by CRISPR/Cas9 in A549 cells. (I) The protein expression levels of TXNRD1, cleaved Caspase 3, Caspase 3, cleaved PARP, and the total form of PARP were determined by Western blotting by CRISPR/Cas9 in A549 cells.

**Figure 4.**
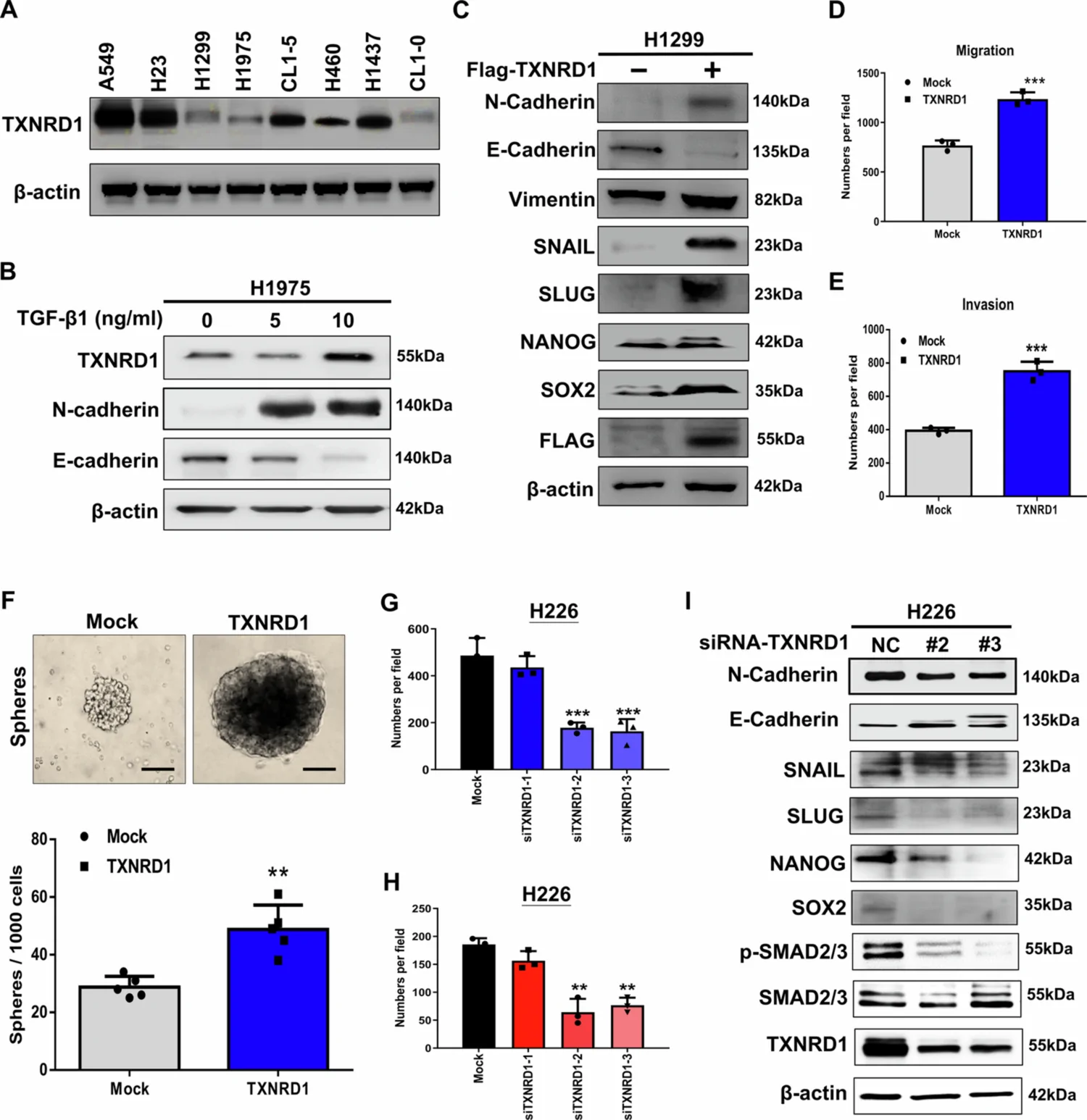
TXNRD1 elevates EMT and stemness-associated phenotypes in NSCLC cells. (A) Western blot analysis of endogenous TXNRD1 expression in eight lung cancer cell lines. *β*-actin is used as a loading control. (B) Western blot analysis of TXNRD1 and EMT markers in cells under TGF-β1 stimulation (0, 5, and 10 ng/mL) in H1975 cells. Whole-cell lysates were subjected to immunoblotting for TXNRD1, *N*-cadherin, and E-cadherin, with *β*-actin as a loading control. (C) Immunoblot analysis of EMT and cancer stemness markers in H1299 cells transfected with control vector or Flag-TXNRD1. Overexpression of TXNRD1 increases *N*-cadherin and decreases E-cadherin, and stemness-associated factors were examined by immunoblotting. *β*-actin serves as a loading control. (D and E) Quantification of cell migration (D) and invasion (E) in Transwell assays comparing mock-transfected and TXNRD1-overexpressing H1299 cells. (F) Representative phase-contrast images (upper) and graphic quantifications (bottom) of spheroid formation in H1299 cells expressing a control vector or TXNRD1. TXNRD1 overexpression leads to larger and more compact spheroids. Scale bars, 20 μm. (G and H) Quantification of cell migration (G) and invasion (H) in Transwell assays comparing siRNA negative control (Mock) and the indicated siTXNRD1-transfected NCI-H226 cells. (I) Immunoblot analysis of EMT, stemness markers, and phospho-SMAD2/3 in NCI-H226 cells transfected with siRNA negative control (NC) or two designed siRNAs for the TXNRD1 gene (human). *β*-actin serves as a loading control. All data are presented as the mean ± SD from independent experiments; statistical significance was calculated versus the corresponding mock control.

To assess the tumorigenic effect of TXNRD1 in NSCLC cells, we performed the Western blot analysis of endogenous TXNRD1 expression in eight lung cancer cell lines (Figure 4A). The specific knockdown of TXNRD1 with CRISPRi sgRNA was performed in A549 cells, which endogenously expressed a high level of TXNRD1. Western blot analysis confirmed efficient TXNRD1 deletion compared with that in the wild-type (WT) controls (Figure 3A). TXNRD1-deficient cells showed significantly reduced migratory (Figure 3B) and invasive (Figure 3C) capacities, suggesting that TXNRD1 deletion significantly reduces tumor cell motility. Time-lapse tracking revealed impaired migratory behavior, characterized by shorter trajectories (Figure 3D), reduced displacement per cell (Figure 3E), and a slower average migration speed (Figure 3F). Additionally, TXNRD1 knockdown markedly reduced tumorsphere formation, indicating diminished cancer stemness (Figure 3G). Notably, previous studies have shown that TXN may play a role in TGF-*β*-mediated EMT-induced tumor mobility and invasion in SACC while also increasing the expression of TXNRD1.^23^ We postulated that TXNRD1 depletion reversed EMT and stemness features at the molecular level. Western blot analysis revealed increased E-cadherin expression and decreased levels of *N*-cadherin, Vimentin, Snail, Slug, Nanog, and Sox2 in TXNRD1-deficient cells compared with WT controls (Figure 3H). Consistent with a previous study demonstrating that TXNRD1 inhibition reduces HCC cell growth and promotes apoptosis,^20^ TXNRD1 deletion led to increased cleaved caspase-3 and cleaved PARP, indicating increased apoptotic cell death (Figure 3I).

### TXNRD1 elevates EMT and stemness-associated signatures in NSCLC cells

To investigate the functional role of TXNRD1 in the EMT and stemness phenotypes of lung cancer cells, in H1975 cells, TGF-β1 stimulation (0, 5, and 10 ng/mL) increased TXNRD1 in a dose-dependent manner and coincided with canonical EMT marker changes, including elevated *N*-cadherin and reduced E-cadherin, suggesting that TXNRD1 is associated with EMT induction (Figures 4A and B). In H1299 cells, which expressed low levels of TXNRD1, forced expression of Flag-TXNRD1 increased the mesenchymal marker *N*-cadherin and decreased the epithelial marker E-cadherin. TXNRD1 overexpression also upregulated the EMT transcription factors SNAIL and SLUG, together with the stemness-associated factors NANOG and SOX2, indicating that TXNRD1 enhances both EMT-related and stem-like molecular features (Figure 4C). Consistent with the EMT-like shift, ectopic expression of TXNRD1 cells exhibited significantly increased migration and invasion compared with mock controls, as quantified by the number of cells per field, supporting a pro-metastatic role of TXNRD1 (Figure 4D and E). Under sphere-forming conditions, TXNRD1 overexpression produced larger and more abundant spheres, with a significant increase in sphere numbers per 1,000 cells relative to that of the controls, reinforcing the association between TXNRD1 and stemness-like phenotypes (Figure 4F). Of note, in human squamous cancer cells (NCI-H226), TXNRD1 knockdown by siRNA reduced the migration (Figure 4G) and invasion ability (Figure 4H). Western blots were performed to determine the expression of EMT-related and cancer stemness-related markers. Compared with the cells transfected with control siRNA, the expression levels of EMT and stemness markers were significantly decreased in H226 cells. In addition, phosphorylated SMAD2/3 levels were reduced upon TXNRD1 silencing without an obvious change in total SMAD2/3, suggesting that TXNRD1 may potentiate TGF-β/SMAD signaling to drive EMT and stemness-associated phenotypes (Figure 4I). Taken together, these findings suggest that TXNRD1 may potentiate TGF-β/SMAD signaling to drive EMT and stemness-associated phenotypes.

### TXNRD1 modulates TGF-β1 signaling and autocrine function in NSCLC cells

**Figure 5.**
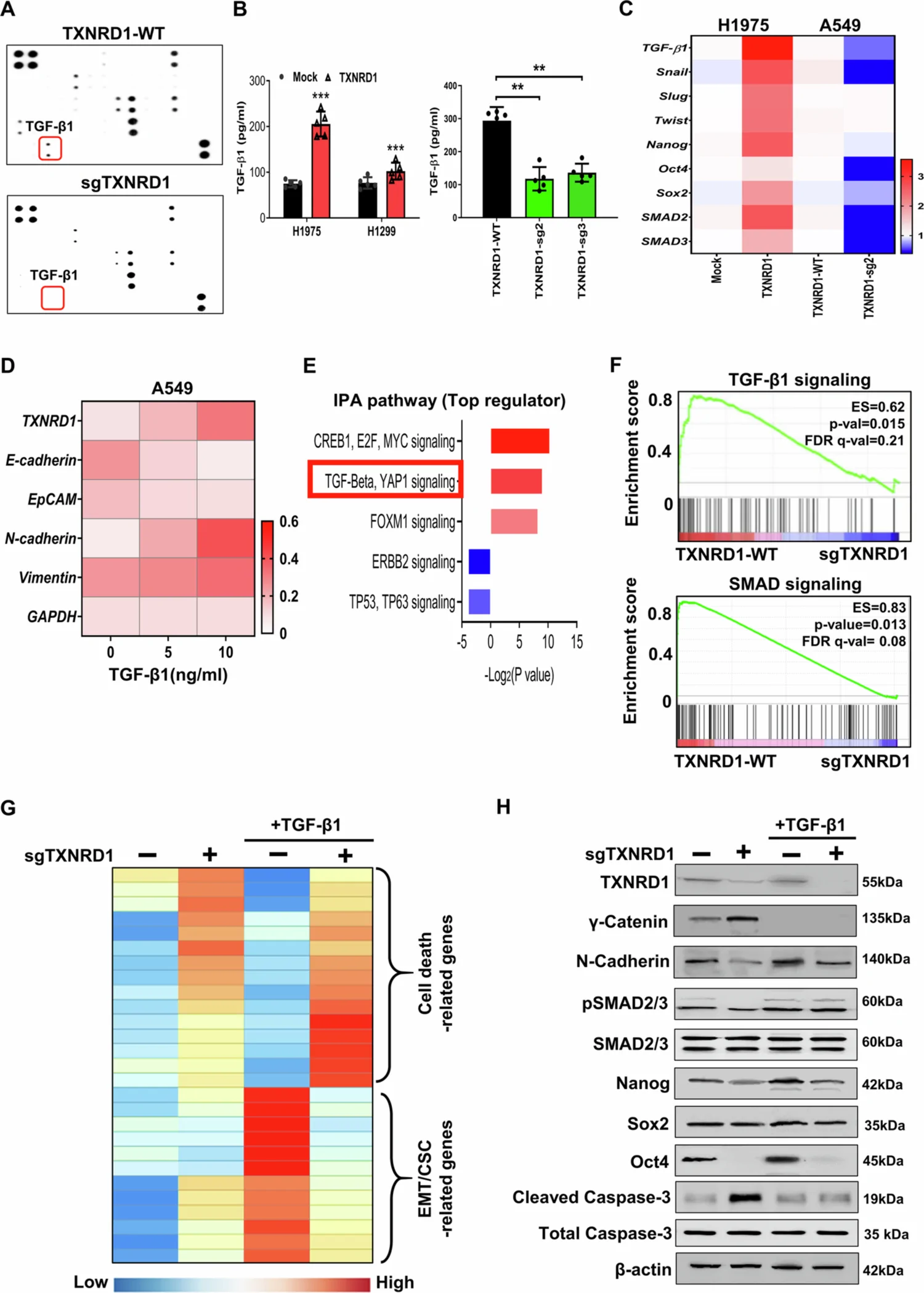
TXNRD1 modulates TGF-β1 signaling and autocrine function in NSCLC cells. (A) Growth factor analysis of TXNRD1-WT and TXNRD1-sg2 groups in A549 cells. Forty-one growth factors were detected, and the significantly changed factors were graphed (red box denotes the TGF-β1 factors). (B) Secreted cytokine TGF-β1 was measured by ELISA assays in the concentrated conditioned medium of H1975 and H1299 cells transfected with TXNRD1 expression constructs and CRISPR/Cas9 knockdown of TXNRD1 in A549 cells. Data represent the mean ± SEM (*n* = 5). All data statistics were based on *p < 0.05, **p < 0.01, and ***p < 0.001 calculated by one-way ANOVA with the Brown–Forsythe test. (C) A heatmap plot of the relative expression profiles of the TGF-β1 downstream signaling pathway in H1975 and A549 cells was detected by qRT-PCR. (D) A heatmap plot of the relative expression profiles of the TXNRD1 and EMT marker signaling in TGF-β1-induced A549 cells (0, 5, or 10 ng/ml for 48 h) was detected by qRT-PCR. (E) Ingenuity pathway analysis (IPA) of the top upstream regulators. Bar chart depicting the top predicted upstream regulators and signaling pathways based on RNA-seq data. The y-axis lists the primary regulatory networks identified (including CREB1/E2F/MYC, TGF-β/YAP1, FOXM1, ERBB2, and TP53/TP63). Red bars extending to the right (positive values) denote predicted pathway activation, while blue bars extending to the left (negative values) denote predicted pathway inhibition. (F) Results of GSE After RNA-sequencing analysis for hallmark analysis, enriched gene sets were shown after transcriptome analysis with the TGF-β1 and SMAD genes signaling origin from TXNRD1-WT and the knockdown of sgTXNRD1 in A549 cells. (G) Heatmap of gene expression based on the intensity of TXNRD1-WT and sgTXNRD1 with/without TGF-β1 induction in A549 cells. The genes are listed on the y-axis, and the conditions are shown on the x-axis. The color scale at the bottom provides a guide to interpret the expression levels, with the range of colors from blue (low) to red (high). This type of plot is commonly used to identify patterns of gene expression, such as genes that are consistently up-regulated or down-regulated in matched gene sets, respectively. (H) Western blotting analysis of the indicated EMT and cancer stemness markers by CRISPR/Cas9 with/without 10 ng/ml TGF-β1 for 48 h in A549 cells.

To determine whether TXNRD1 regulates growth factor secretion, we performed growth factor array analysis in A549 cells. Among the 41 factors, several were differentially expressed between TXNRD1-WT and TXNRD1-deficient (sgTXNRD1) cells, with TGF-β1 emerging as one of the most significantly downregulated factors following TXNRD1 loss (Figure 5A). The protein array data for the sgTXNRD1-2 group, which exhibited a trend similar to that of the sgTXNRD1-3 group (data not shown). This observation was validated by ELISA, which demonstrated that TXNRD1 overexpression significantly increased extracellular TGF-β1 secretion in H1975 and H1299 cells, whereas TXNRD1 knockdown reduced TGF-β1 levels in A549 cells (Figure 5B). To determine whether TXNRD1 regulates TGF-β1 production at the transcriptional level, we performed a TGF-β1 promoter luciferase activity assay using the pGL3-TGF-β1 reporter construct from our previous study.^24^ ^,^ ^25^ As shown in Supplementary Figure 1A, TXNRD1 depletion significantly reduced TGF-β1 promoter-driven luciferase activity compared to TXNRD1-WT cells. However, we analyzed public ChIP-seq datasets to distinguish direct NRF2-mediated regulation from TXNRD1-associated regulation of the TGF-β1 promoter. NRF2/NFE2L2 and small MAF factors showed strong occupancy at TXNRD1 but not at the TGF-β1 promoter, suggesting that NRF2 directly regulates TXNRD1, whereas TGF-β1 promoter modulation is more likely to be mediated indirectly through TXNRD1-dependent redox-sensitive transcriptional mechanisms (Supplementary Figure 1B). Transcriptomic analysis showed that TXNRD1 deletion reduced TGF-β1-induced global gene expression alterations in both H1975 and A549 cells (Figure 5C). In A549 cells, TGF-β1 treatment activated an EMT program characterized by increased *N*-cadherin and Vimentin expression and decreased E-cadherin and EpCAM levels (Figure 5D). However, TXNRD1 deficiency significantly reduced TGF-β1 expression (Figure 5C). These results implied that TXNRD1 not only modulates TGF-β1 downstream signaling but also regulates its extracellular availability, rather than causing a uniform change in all secreted factors, supporting a specific regulatory link between TXNRD1 and TGF-β1 signaling. RNA-seq followed by Ingenuity Pathway Analysis (IPA, Qiagen) identified TGF-β/YAP1 signaling was substantially altered upon TXNRD1 knockdown (Figure 5E). Gene set enrichment analysis (GSEA) further confirmed lower activation of canonical TGF-β/SMAD signaling in TXNRD1-deficient cells (Figure 5F). Heatmap analysis demonstrated that TGF-β1 robustly induced EMT- and stemness-associated gene sets in control cells, including cell adhesion molecules, extracellular matrix regulators, and pluripotency-related transcription factors, which were markedly attenuated in TXNRD1-knockdown cells (Figure 5G). Of note, we performed TGF-β1 induction with TXNRD1-knockdown A549 cells (sgTXNRD1) treated with exogenous recombinant TGF-β1 for 48 h. As shown in Figure 5H, exogenous TGF-β1 rescued the expression of mesenchymal markers (*N*-cadherin, vimentin) and stemness factors (nanog, Sox2, Oct4) that were suppressed by TXNRD1 deletion, as well as SMAD2/3 phosphorylation, suggesting that TXNRD1 loss impairs EMT/stemness primarily through TGF-β1 availability, directly supporting the autocrine loop model. These results indicate that TXNRD1 functions upstream by regulating SMAD2/3 activation and preserving the expression of downstream transcriptional networks that promote cellular plasticity, as demonstrated by the decrease in both EMT and stemness-related proteins. Collectively, these results suggest that TXNRD1 is an essential regulator of TGF-β1 signaling, which is vital for NSCLC plasticity, EMT induction, and the acquisition of stem-like characteristics.

### Loss of TXNRD1 delays tumor growth in an orthotopic lung cancer model.

**Figure 6.**
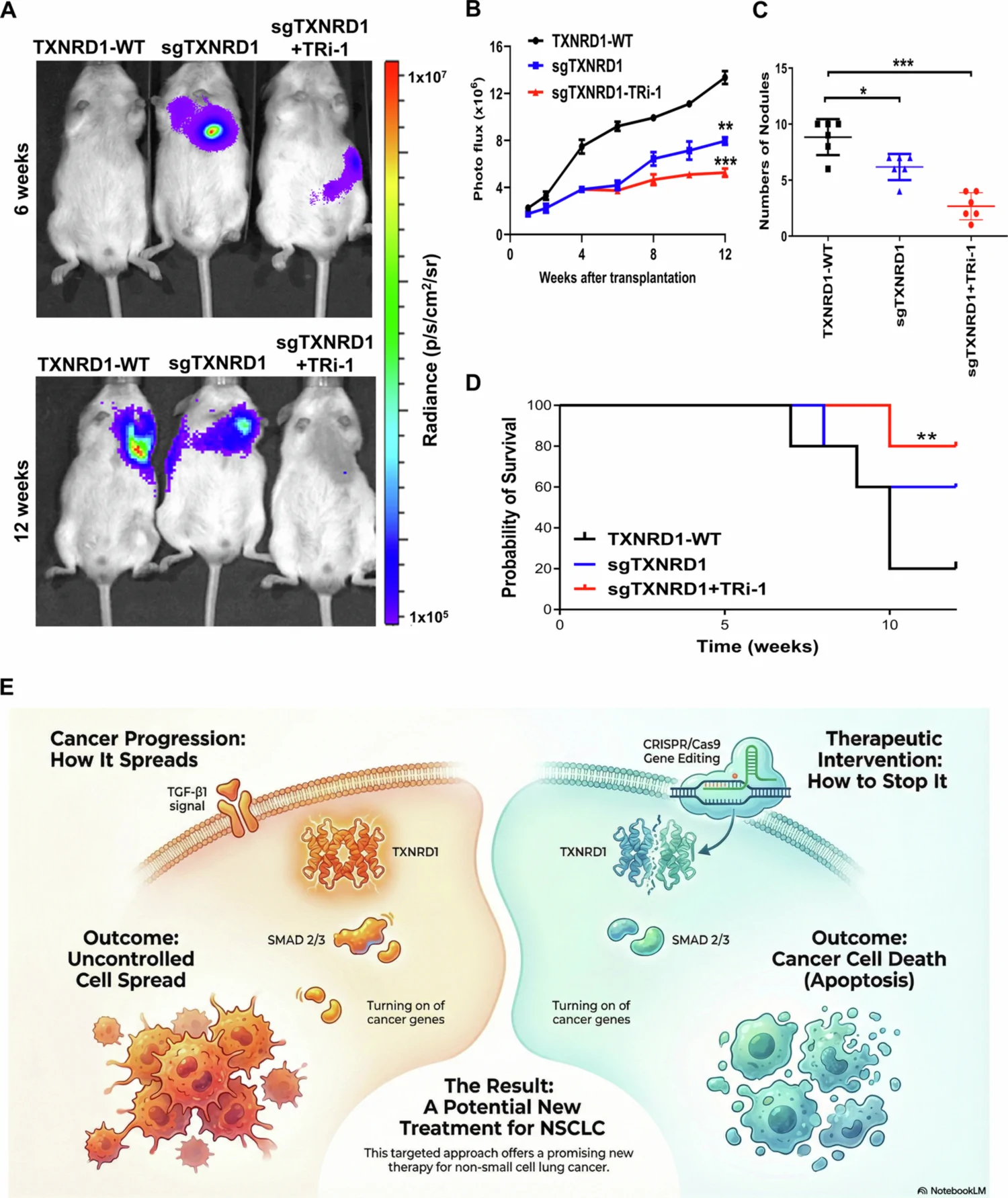
Loss of TXNRD1 impaired metastatic tumor growth in an orthotopic model of lung cancer. (A) Representative bioluminescence imaging of lung colonization in mice injected with TXNRD1-WT cells, TXNRD1-sg cells, and TXNRD1-sg cells treated with a TRi-1 inhibitor at 6 and 12 weeks after transplantation. The color scale indicates the photon flux intensity from low (purple) to high (red). (B) Quantitative analysis of the bioluminescence signal intensity over time (mean ± SEM, *n* = 5/group). (C) Tumor nodules were measured at the experimental endpoint after cell transplantation. (D) Kaplan–Meier survival curves comparing the TXNRD1-WT (black), TXNRD1-deficiency group (blue), and TXNRD1-deficiency plus TRi-1 treatment (red) groups in an orthotopic model of lung cancer (*n* = 5/groups). Statistical significance was determined by Student’s t-test or log-rank test; *p* < 0.05 was considered significant. (E) A schematic representation depicting the role of transforming growth factor-β1 (TGF-β1) signaling in facilitating epithelial–mesenchymal transition (EMT) and the production of cancer stem cell (CSC)-like genes in non-small cell lung cancer (NSCLC). Upon binding to the TGF-*β* receptor (TGF-βR), TGF-β1 activates thioredoxin reductase 1 (TXNRD1), thereby enhancing the phosphorylation of SMAD2/3 and promoting the transcription of EMT/CSC-related genes, resulting in epithelial cells adopting a mesenchymal phenotype. The CRISPR/Cas9-induced deletion of TXNRD1 inhibits this pathway, disrupting EMT/CSC gene activation and thereby enhancing apoptosis in cancer cells and delaying tumor progression in NSCLC. This figure was generated and exported using Google NotebookLM. The image bears the “NotebookLM” watermark in the bottom-right corner, indicating that it was generated by NotebookLM's built-in AI visualization workflow using the inputs provided within the NotebookLM environment.

To evaluate the physiological consequences of TXNRD1 depletion in vivo, we performed orthotopic lung colonization assays using luciferase-labeled cancer cells. Mice were injected with TXNRD1-WT cells or TXNRD1-deficient cells or treated with TXNRD1 inhibitor 1 TRi-1, which specifically binds to TXNRD1 and inhibits TXNRD1 activity *in vivo*.^21^ ^,^ ^26^ ^,^ ^27^ In this study, we injected human A549 cells infected with luciferase-containing cells monitored by a bioluminescent IVIS imaging system directly into the left pleural cavity of NOD/SCID mice. As expected, TXNRD1-WT and sgTXNRD1 in A549 cells proliferated significantly within the pleural cavity. All the indicated cell lines in the subcutis of NOD/SCID mice were successfully engrafted in the pleural cavity. Bioluminescence imaging revealed significant tumor growth in the TXNRD1-WT group, as shown by robust luminous signals representing tumor development. In contrast, the TXNRD1-deficiency plus TRi-1–treated group exhibited substantially reduced luminescence intensity, suggesting effective suppression of tumor growth (Figure 6A). Quantification of total photon flux confirmed a significant reduction in signal intensity in the treatment group compared to the control group from day 14 onward (Figure 6B). The tumor nodule counts consistently showed reduced tumor growth rates in the treatment groups (Figure 6C). Kaplan–Meier survival analysis revealed prolonged survival in the treatment group compared to controls, indicating that the therapy not only reduced the growth of tumors but also prolonged overall survival (Figure 6D). Collectively, these findings suggested that TXNRD1 is involved in TGF-β1-induced EMT in NSCLC cells. TGF-β1 stimulation elevated TXNRD1 expression and SMAD2/3 phosphorylation, activating genes associated with EMT and cancer stem cells. TXNRD1 deletion inhibited EMT and triggered cell death in NSCLC cells, revealing its role in TGF-β1-driven EMT and CSC features. Therefore, TXNRD1 acts as a critical downstream effector of TGF-*β* signaling, promoting EMT and CSC-like features in NSCLC, and its inhibition induces apoptotic cell death, as demonstrated in Figure 6E for the proposed model.

## Discussion

In this study, we identified TXNRD1 as a critical regulator in the progression and recurrence of NSCLC. TXNRD1 expression strongly correlates with clinical stage, highlighting its potential as a prognostic biomarker. Mechanistically, TXNRD1 promotes cellular motility and cancer stemness—hallmarks of metastatic competence—by sustaining TGF-β1 autocrine signaling and downstream EMT programs. Importantly, genetic deletion or pharmacological inhibition of TXNRD1 significantly reduced metastatic growth in an orthotopic lung cancer model, underscoring its critical role in tumor progression *in vivo*. TXNRD1 may serve as both a predictive biomarker and a potential therapeutic target. Combinations with established strategies such as immune checkpoint blockade or targeted therapy warrant further investigation. Notably, our findings indicated that TXNRD1 expression was significantly associated with NSCLC stage progression and tumor recurrence. Unlike oncogenic mutations such as *EGFR* or *KRAS*, which are restricted to specific subsets of patients with lung cancer, TXNRD1 overexpression appears broadly prevalent across NSCLC cases.^28^ Its strong association with poor clinical outcomes suggests that TXNRD1 may complement existing staging systems, such as TNM classification. Furthermore, evaluating TXNRD1 expression may aid in clinical decision-making by identifying patients at a high risk of early recurrence, developing biomarker panels, and improving predictive accuracy.

While previous studies have identified other antioxidant enzymes, such as glutathione peroxidase (GPXs) and superoxide dismutase (SODs), are frequently dysregulated in cancers,^29^ ^,^ ^30^ *TXNRD1* expression exhibited a strong and consistent correlation with NSCLC progression and recurrence, underscoring its superior prognostic significance. Our analysis highlights the distinct clinical and prognostic significance of TXNRD1 by identifying it as the most significant stage progression-related gene in NSCLC. Mechanistically, we found that TXNRD1 promoted NSCLC aggressiveness by increasing cell motility and stemness, indicating that TXNRD1 may be a robust indicator of metastatic competence. TXN affects SACC invasion and metastasis by modulating TGF-*β*-Akt/GSK-3β signaling during EMT. Thus, TXN may be a possible treatment target for SACC.^23^ A limitation of this study is that, although our data support an association between TXNRD1 and activation of the TGF-β/SMAD pathway, we did not directly experimentally validate that TXNRD1-mediated redox changes causally drive TGF-β1 production or SMAD2/3 phosphorylation. Future studies will determine whether manipulating the cellular redox status downstream of TXNRD1 (e.g., the ROS/GSH balance) directly regulates TGF-β1 levels and SMAD2/3 activation. Notably, our study revealed that TXNRD1 regulates TGF-β1 signaling, a crucial mechanism for tumor growth and immunological modulation, resulting in autocrine TGF-β1 activation that fosters an environment conducive to invasion and metastasis.

As a thioredoxin reductase, TXNRD1 can also interact with oxidative stress pathways. TXNRD1 may connect redox regulation to pro-metastatic signaling pathways. Previous studies have demonstrated that thioredoxin reductases facilitate EMT and confer resistance to oxidative stress in aggressive cancers.^31^ TGF-β1 signaling is a key regulator of EMT and metastatic spread in NSCLC.^32^ Notably, the activation of antioxidant pathways is generally regarded as a key event during tumorigenesis, as tumor cells require antioxidants to buffer excessive ROS levels and avoid severe oxidative damage.^33^ Other enzymes, such as GPXs and SODs, also detoxify ROS and maintain redox balance. Their significance in cancer biology is primarily indirect, and their prognostic or mechanistic contributions are context-dependent and often contradictory across cancer types. GPX2 overexpression promotes EMT and tumor development in lung cancer and castration-resistant prostate cancer.^34^ ^,^ ^35^ Similarly, SOD2 decreases breast cancer risk while promoting prostate and salivary adenoid cystic carcinomas.^36^ ^,^ ^37^ In contrast, TXNRD1 consistently promotes oncogenic signaling in various cancer types, including liver, breast, and lung malignancies,^38-40^ with particularly strong effects in the NSCLC clinical cohort. TXNRD1, unlike GPX and SOD, maintains TGF-β1-mediated autocrine signaling and promotes cancer stemness. Therefore, our study highlights TXNRD1 as a uniquely dependent driver of NSCLC aggressiveness, distinguishing it from other antioxidative enzymes.

Thioredoxin reductases are composed of three isoforms with different cellular localizations and biological functions: TXNRD1 (cytosolic), TXNRD2 (mitochondrial), and TXNRD3 (testis-enriched), suggesting that these isoforms perform distinct and sometimes competing roles in various malignancies.^38^ TXNRD2 has been linked to the maintenance of mitochondrial redox equilibrium and protection against apoptosis.^39^ ^,^ ^40^ While some studies have shown that it has tumorigenicity in glioblastoma and gastric cancer, others have shown anti-tumor activity in hematological malignancies, emphasizing its context-dependence. TXNRD3 is predominantly expressed in the testis and reproductive tissues, with limited evidence supporting its role in cancer, whereas sporadic studies suggest small contributions to germ cell-associated cancers.^40^ This inter-isoform heterogeneity is crucial for clinical translation. TXNRD2 and TXNRD3 exhibit tissue-specific or cancer-type-specific activities, whereas TXNRD1 displays a consistent pro-oncogenic profile across various tumor types.

The molecular mechanism by which TXNRD1 sustains TGF-β1 availability operates at the transcriptional level through redox-sensitive signaling. TXNRD1 could support the activity of redox-sensitive transcription factors, including NRF2 and activator protein-1 (AP-1).^41^ NRF2 has been shown to bind antioxidant response elements (AREs) within the TGF-β1 gene promoter region, thereby promoting its transcriptional activation under conditions of oxidative adaptation in cancer cells.^42^ Consistent with these findings, our data demonstrated that TXNRD1 knockdown reduced TGF-β1 mRNA levels (Figure 5C), suggesting that TXNRD1 loss destabilizes NRF2 activity and consequently attenuates TGF-β1 transcription. Moreover, the dual role of TXNRD1 in both supporting TGF-β1 transcription (via NRF2/AP-1 stabilization) and potentially modulating latent TGF-β1 activation may thus explain why TXNRD1 overexpression robustly increases extracellular TGF-β1 levels, as observed in our ELISA data (Figure 5B). Once secreted, TGF-β1 phosphorylates SMAD2/3, which we demonstrated is suppressed upon TXNRD1 deletion (Figures 4 and 5), thereby disrupting the downstream transcription of EMT effectors and stemness factors.^43^ However, we understand a significant limitation: the current study lacks direct experimental evidence – such as the use of TGF-β1 promoter-luciferase reporter assays or chromatin immunoprecipitation for NRF2/AP-1 binding at the TGF-β1 locus to establish a relationship between TXNRD1-mediated redox alterations and TGF-β1 transcription. Future research utilizing site-directed redox perturbations (e.g., pharmacological activation of NRF2 or AP-1 dominant-negative constructs in TXNRD1-knockout) will be essential to clarify the specific transcriptional hierarchy by which TXNRD1 regulates TGF-β1 production in NSCLC.

Our study suggests that TXNRD1 in NSCLC not only predicts advanced disease and recurrence but also promotes metastasis by sustaining TGF-β1-mediated autocrine signaling and cancer stemness. The therapeutic potential of TXNRD1 is promising. Our in vivo study showed that TXNRD1 deletion reduced metastatic tumor growth, confirming its role as a functional biomarker. Importantly, TXNRD1 is particularly attractive because of its enzymatic druggability. Several thioredoxin reductase inhibitors, including auranofin, have demonstrated anticancer activity in preclinical models.^44^ ^,^ ^45^ Furthermore, combining TXNRD1 inhibition with immune checkpoint inhibitors or tyrosine kinase inhibitors could lead to synergistic results by modulating redox balance and tumor immune evasion.

## Conclusion

Our study establishes TXNRD1 as a central link between redox regulation and metastatic signaling in NSCLC. TXNRD1 functions as both a prognostic biomarker and a mechanistically validated target, highlighting a strong rationale for its clinical evaluation in NSCLC.

## Materials and Methods

### Cell lines and culture reagents

The human NSCLC cell lines A549 (CCL-185), H1299 (CRL-5803), H1975 (Cytion™, 305067, Germany), H226 (Cytion™, 305091, Germany) were obtained from the American Type Culture Collection (ATCC), and Cytion™ were cultured in Dulbecco's modified Eagle medium (DMEM, Gibco-BRL,11965092, USA), and RPMI 1640 (Corning, 10-040-CV, USA), supplemented with 10% fetal bovine serum (FBS, Gibco-BRL, 10270106, USA), penicillin–streptomycin (Gibco-BRL, 15140122, USA), and 2 mM L-glutamine (Gibco-BRL, 25030081, USA) in 5% CO_2_ at 37 °C. The TXNRD1 gene was amplified from NL20 (immortalized lung epithelial cell) cDNAs and cloned into the pcDNA3.1-Flag vector. The constructs were introduced into H1299 and H1975 cells, and stable clones were generated by neomycin selection (Sigma-Aldrich, A1720, USA). Empty vector-transfected cells were used as controls. All the cell lines were maintained in a humidified incubator at 37 °C supplemented with 5% CO₂. For the mycoplasma control, the cell cultures were treated with Plasmocin® Mycoplasma Elimination Reagent (InvivoGen, ant-mpp, USA) according to the manufacturer’s instructions. Plasmocin prophylactic (2.5 mg/ml) was routinely added to the liquid media to prevent mycoplasma contamination in all the cell cultures. Following treatment, mycoplasma contamination was assessed by a PCR-based mycoplasma test, and only mycoplasma-negative cultures were used for experiments.

### In silico analysis of TXNRD1 expression in TCGA datasets

Transcriptome datasets from TCGA (level 3 RNA-seq V2) were downloaded from the UCSC Xena browser (https://ucsc-xena.gitbook.io/project/public-data-we-host/tcga) in April 2021. RNA expression values for tumor and adjacent normal tissues from patients with NSCLC were obtained from the HiSeqV2_PANCAN datasets. Redox system-related genes included members of the TXN, SOD, and GPX families. The expression levels in lung adenocarcinoma and lung squamous cell carcinoma were visualized graphically in TCGA (https://tcga-data.nci.nih.gov/tcga/). Expectation-maximization (RSEM) values extracted from RNA-seq data were used for downstream analysis of TXNRD1 expression across different cancers. Normalized counts were analyzed using two-sided t-tests and log2 fold changes to compare the target groups in a pairwise fashion. Ten datasets of NSCLC T-stage samples were downloaded from the GEO dataset (GSE14108, GSE14814, GSE27716, GSE30219, GSE31210, GSE33532, GSE31546, GSE37745, GSE43580, and GSE68465) with T-stage (primary tumor) information. *TXNRD1* expression levels were normalized across different T-stage primary tumors and metastasized distant organ samples.

### Data sources and cohort analysis of TXNRD1

Publicly available transcriptomic and clinical data were analyzed. The TCGA cohort (*N* = 482) was obtained from The Cancer Genome Atlas (TCGA). Independent microarray cohorts were retrieved from the Gene Expression Omnibus (GEO), including GSE68495 (*N* = 442), GSE50081 (*N* = 128), GSE37745 (*N* = 107), GSE30219 (*N* = 86), and GSE14814 (*N* = 42). In addition to single-cohort analyses, these GEO cohorts were combined to form a pooled microarray dataset (*N* = 801) after batch-effect adjustment. TXNRD1 expression values were retrieved from each dataset. To ensure comparability and eliminate deviations, TXNRD1 expression was measured using a log2 scale (TXNRD1 log2). Probes in microarray datasets were mapped to gene symbols using platform annotations; when numerous probes mapped to TXNRD1, a single representative probe was chosen using a consistent rule (e.g., highest average expression across samples) to avoid repeating gene-level measurements.

### Survival analysis and multivariable modeling

The Cox proportional hazards regression model was used to investigate the associations between TXNRD1 expression and OS. TXNRD1 (log2) was used as a continuous predictor. Multivariable Cox regression analyses were performed to test the prognostic value of TXNRD1 expression after adjusting for TNM stage, age, and sex. The results are reported as hazard ratios (HRs) with 95% confidence intervals (CIs). Two-sided tests were used to determine statistical significance, and a *p*-value < 0.05 was considered significant. Standard diagnostics, such as Schoenfeld residual-based tests and/or visual inspections, were used to evaluate the proportional hazards assumption where applicable. All analyses were performed in the R statistical environment, including Cox regression, as well as typical data wrangling and visualization tools.

### Preparation of TXNRD1 gRNA and RNP complexes

CRISPR guide RNA (gRNA) targeting exon 6 of the TXNRD1 gene (gRNA-1: CACCCCTCTTGGAACTAGAT-GGG, gRNA-2: CTGCAAGACTCTCGAAATTA TGG cut site: chr6 [+104313294~5853], NM_001093771.3 → NP_001087240.1) was designed using an online tool from the Integrated DNA Technologies (IDT, Coralville, USA). The sgRNAs of TXNRD1 were manually modified to match the mutant sequence in question and verified using the IDT CRISPR-Cas9 guide RNA design checker (https://eu.idtdna.com/site/order/designtool/index/CRISPR_SEQUENCE). Ribonucleoprotein (RNP) complexes were synthesized according to the Alt-R CRISPR-Cas9 System protocol. Equimolar amounts of gRNA and SpCas9-GFP were mixed to a final concentration of 300 nM in 30 μL of reaction buffer and incubated at room temperature for 10 min. Alt-R S.p. Cas9-GFP V3 nucleases (IDT, 10007924, USA) containing fusion proteins with nuclear localization sequences and a C-terminal 6-His tag were used to facilitate post-transfection visualization or fluorescence-activated cell sorting (FACS).

### RNP transfection and sorting

A549 cells were cultured in DMEM supplemented with 10% FBS and maintained in 5% CO₂.. Transfection of A549 cells was performed using Lipofectamine™ CRISPRMAX (Thermo Fisher Scientific, CMAX00003, USA) according to the manufacturer’s instructions. The A549 GFP-transfected cell line was obtained, subcloned, and sorted by flow cytometry (FACS) using a BD LSR cell analyzer. A549 cells seeded in 96-well plates were pelleted by centrifugation at 1000 × g for 1 min and resuspended in 500 μL of FACS buffer containing 1% bovine serum albumin in phosphate-buffered saline. A total of 10,000–20,000 GFP-positive cells were analyzed. Untreated cells served as negative controls. FACS data were analyzed using FlowJo_v10.8.1 software (Ashland, USA).

### Cell migration, invasion, and live-cell time-lapse imaging

Transwell migration and invasion assays were conducted as previously described.^24^ Cells seeded in the upper chamber in 8 μm polyethylene terephthalate, Millicell Cell Culture Inserts (Millipore, PTEP24H48, USA), were allowed to migrate to the lower membrane surface. They were subsequently fixed with methyl alcohol and stained with 0.5% crystal violet for 30 min each. The migrated cells were photographed in at least five random fields per well using a Nikon fluorescence microscope. Cell numbers were counted automatically using the “Analyze Particles” function in ImageJ. For live-cell time-lapse imaging, cells were seeded into 24-well plates at 80% confluence. The migrated cells were photographed every 30 mins for 24 h using a Leica DMI 6000 B microscope and analyzed using MetaMorph image analysis software. All the assays were conducted in triplicate.

### RNA isolation and RT-qPCR

Total RNA was extracted using TRIzol (Thermo Fisher Scientific, 15596026, USA) according to the manufacturer's instructions. The RNA was reverse-transcribed into cDNA using oligo-dT primers and a First-Strand cDNA Synthesis kit (Thermo Fisher Scientific, K1612, USA). Reverse transcription quantitative polymerase chain reaction (RT-qPCR) was performed using SYBR Green PCR Master Mix (Thermo Fisher Scientific, 4309155, USA) on an Applied Biosystems 7500 Real-Time PCR System. *β*-actin served as the internal control for normalization. The primer sequences used for RT-qPCR analysis of the TGF-β1-related genes are listed in Supplementary Table 1.

### siRNA-based Knockdown of TXNRD1

Human lung squamous cell carcinoma NCI-H226 cells were seeded in 6-well plates and transfected with either the TXNRD1 Human Pre-designed siRNA Set A or the non-targeting control siRNA (MedChemExpress, HY-RS15298, USA) at a final concentration of 50 nm using jetPRIME® (Polyplus-transfection, 101000046, France), following the protocol. After 48 h of successful transfection, the cells were harvested for subsequent assays.

### Western blot analysis

Total protein was extracted using RIPA buffer containing protease (Roche, 11836153001, Switzerland) and phosphatase inhibitors (Roche, 04906845001, Switzerland). The protein concentration was quantified using the Bradford assay kit (BioRad, 5000006, USA). Equal amounts of protein were loaded and separated by 10–12% SDS–PAGE and transferred to PVDF membranes (Millipore, IPVH00010, USA). The membranes were incubated with primary antibodies overnight at 4 °C, followed by incubation with HRP-conjugated mouse and rabbit secondary antibodies (1:5000) for 1 h at room temperature. Protein bands were detected using a ChemiDoc XRS + system (Bio-Rad). Primary antibodies against TXNRD1, EMT, and cancer stem cell (CSC)-related genes (diluted 1:1000) were purchased from ABclonal: TXNRD1 (A4725) and Cas9 (A23005). Cell Signaling Technology: E-Cadherin (24E10), *N*-cadherin (D4R1H), Vimentin (D21H3), Snail (C15D3), Slug (C19G7), Nanog (D73G4), Sox2 (D6D9), Oct-4 (D7O5Z), Caspase-3 (D3R6Y, 14220), Cleaved Caspase-3 (Asp175 9661), PARP (46D11, 9532), Cleaved PARP (Asp214, 5625), SMAD2/3 Antibody Sampler Kit (12747), and *β*-actin (1:10000, A5441, Sigma–Aldrich, USA). Goat anti-mouse IgG-horseradish peroxidase (HRP) and goat anti-rabbit IgG-HRP were used at a 1:5000 dilution as secondary antibodies (Thermo Fisher Scientific, 31430 and 31460, USA).

### TGF-β1 promoter luciferase reporter assay

Control and TXNRD1-knockdown cells were seeded in 24-well plates and co-transfected with the pGL3-TGF-β1 promoter reporter plasmid and a Renilla luciferase plasmid as an internal normalization control.^25^ After 24 h, firefly and Renilla luciferase activities were measured using the Dual-Luciferase Reporter Assay System (Promega, E2940, USA) according to the manufacturer’s instructions. TGF-β1 promoter activity was calculated by normalizing firefly luciferase activity to Renilla luciferase activity. Data are presented as mean ± SD from at least three independent experiments.

### Public ChIP-seq data mining

To assess transcription-factor occupancy at the TGF-β1 and TXNRD1 loci, we mined publicly available ChIP-seq data from ChIP-Atlas (https://chip-atlas.dbcls.jp) and the ENCODE portal (https://www.encodeproject.org), using the human genome assembly GRCh38/hg38. Gene coordinates were obtained from Ensembl: TGF-β1 (ENSG00000105329) spans chr19:41,301,587–41,353,961 on the minus strand, with the TSS at chr19:41,353,961. ChIP-Atlas target-gene tables (antigen class “TFs and others,” MACS2 Q < 1 × 10^−5^, TSS ± 1 kb) provided, per transcription factor, the averaged binding score and the number of supporting experiments, including the lung adenocarcinoma line A549. For peak-level mapping (Panel A), narrowPeak BED files for the top-scoring A549 experiments (JUN, JUNB, JUND, FOSL2, SP1) were downloaded and intersected with strand-aware promoter windows derived from the TSS: the AP-1/TRE + Sp1 second-promoter zone (chr19:41,353,690–41,353,960) and the −454/−323 autoinduction region (chr19:41,354,284–41,354,415). AP-1/Sp1 occupancy was independently confirmed against ENCODE optimal-IDR peaks for A549 cells (JUN, ENCSR604CKQ/ENCFF094MAD; JUND, ENCSR000BRF/ENCFF587VEY; FOSL2, ENCSR448TVS/ENCFF075JEN; SP1, ENCSR000BPE/ENCFF404OSB). The NRF2/small-MAF arm (Panel C) was evaluated by querying NFE2L2, MAFF, MAFG, and MAFK at TXNRD1 versus TGF-β1; absence at TGF-β1 was interpreted relative to each factor's genome-wide target-gene set size (NFE2L2: 8,552 genes). Downstream EMT regulators (Panel D; SMAD3, SMAD4, SNAI1, SNAI2, ZEB1, ZEB2, and TWIST1) were queried against canonical EMT genes (CDH1, CDH2, VIM, FN1, SNAI1, SNAI2, ZEB1, SERPINE1, and TGF-β1); cells lacking individual supporting experiments at TSS ± 1 kb were masked. Data handling and visualization used Python 3.11 with pandas, NumPy, Matplotlib, and seaborn; figures were exported at 300 dpi. The ChIP-seq peaks delineate occupied promoter regions at the resolution of the assay (~150–650 bp) and indicate transcription-factor occupancy rather than TXNRD1-dependence.

### RNA-Seq analysis

RNA-seq was performed as previously described.^24^ Total RNA was extracted using the NucleoSpin RNA kit (Macherey-Nagel, 740955.50, Germany) and assessed for quality using spectrophotometry, agarose gel electrophoresis (determined by the 18S and 28S rRNA ratios), and an Agilent Technologies 2100 Bio-analyzer (Agilent Technologies, 5067-1511, USA). Samples with RNA integrity numbers (RIN) >8 were processed for library preparation. After rRNA depletion, RNA fragmentation, and library preparation (Illumina), the constructed libraries were subjected to 150 bp paired-end sequencing using an Illumina NovaSeq 6000 sequencer at BIOTOOLS (Taiwan).

### Gene set enrichment analysis (GSEA)

GSEA was performed using gene sets from the MSigDB database and published gene signatures. Gene sets with a false discovery rate value <0.05 by comparing the enrichment score to enrichment results generated from 1000 random permutations were considered statistically significant.

### Immunohistochemistry (IHC) staining from a human tissue array

IHC was performed as described previously.^24^ After deparaffinization, the tissue sections were subjected to 10 mM citrate buffer (pH 6.0) and microwave treatment for 20  min for antigen retrieval. The samples were immersed in 3% H₂O₂ for 30 min to block endogenous peroxidase and then incubated with an anti-TXNRD1 primary antibody overnight at 4 °C (1:100, Abcam, EPNCIR129, UK). The slides were processed using a SuperPicture Polymer Detection kit (Invitrogen, 87965, USA) according to the manufacturer’s protocol and then counterstained with hematoxylin. The tissue arrays were purchased from SUPER BIO CHIPS (SuperBioChips Laboratories, CCA-3, Korea). The use of human cancer tissue arrays obtained from commercial sources was approved by the Human Subject Research Ethics Committee of Taipei Medical University (TMU-JIRB: N202404093). All the IHC results and their associations with patient survival were examined and scored from 1 to 4, multiplied by the percentage of stained cells (H-score) based on expression intensity, as assessed by an independent pathologist at Taipei Medical University Hospital. Briefly, staining intensity was scored as unfavorable (score = 0), weakly positive (score = 1), moderately positive (score = 2), or strongly positive (score = 3). The final H-score was calculated using the following equation (range, 0–300): H-score = 0 × (% of cells with a score of 0) + 1 × (% of cells with a score of 1) + 2 × (% of cells with a score of 2) + 3 × (% of cells with a score of 3). Upon evaluation, statistical analysis was performed using GraphPad Prism (v.8.0 USA). The student's t-test was used to compare IHC expression in continuous variables, and categorical variables were assessed using the chi-square test or Fisher’s exact test. Statistical significance was set at *p* < 0.05.

### Tumorsphere formation assay

A549 cells were seeded at 1 × 10^4^ cells/mL in ultralow attachment 24-well plates (Corning, 3473, USA). The cells were cultured in serum-free medium supplemented with B-27 (Gibco-BRL, 17504044, USA), 20 ng/mL basic fibroblast growth factor (bFGF; Elabscience, PKSH032439, China), and epidermal growth factor (EGF; Elabscience, ELABS-R-005, China). Tumorspheres were allowed to form for 7–14 d and were imaged using an optical microscope (Zeiss, Germany).

### Transfections with knockdown or overexpression reagents

*TXNRD1* knockdown in A549 cells was achieved using CRISPR interference (CRISPRi; CRISPR Gene Targeting Core Lab at TMU, Taiwan). sgRNA vectors targeting *TXNRD1* were transfected with p5w-dCas9-KRAB.pPURO plasmids into A549 cells using jetPRIME protocols (PolyPlus, 101000046, France). Stable clones were selected using 2 μg/mL puromycin and 10 μg/mL blastidin (InvivoGen, ant-br-1, USA) after at least 2 weeks. Luciferase expression vectors peIF4g and pMD2. G was transfected into 293FT cells using jetPRIME for virus production. After 2 d, the virus-containing supernatant containing 8 μg/ml polybrene (Sigma–Aldrich, H9268, USA) was collected by passing through 0.22 μm filters, which were then ready for A549 infection. Luciferase activities of firefly and Renilla were measured using the Dual-Glo® Luciferase Assay System (Promega, E2920, USA) based on the manufacturer’s instructions. A549 cells were infected with a luciferase-expressing lentivirus for a lung orthotopic model and selected by 2  μg/ml puromycin (InvivoGen, ant-pr-1, USA). The full-length *TXNRD1* cDNA was subsequently cloned into the pcDNA3.0-FLAG plasmid. Plasmids for the overexpression of TXNRD1 were transfected into cells according to the standard JetPRIME protocol.

### Lung orthotopic mouse models

NOD-SCID/CB.17 mice (6–8 weeks old) were purchased from BioLASCO (Taiwan) and housed in the animal facility of Taipei Medical University. For orthotopic lung implantation, the mice were anesthetized with isoflurane (Sigma–Aldrich, 792632, USA) by induction: 3%; maintenance: 1.5% in oxygen, delivered via a precision vaporizer using an induction chamber followed by a nose cone according to institutional veterinary guidance. A549 luciferase-expressing cells (1 × 10^6^ cells in 50 μL) were mixed 1:1 (v/v) with Matrigel (BD Biosciences, 354234, USA) and injected into the left lung as described previously.^46^ The TXNRD1 inhibitor TRi-1 was purchased from MedChem Express (MedChemExpress, HY-125006, USA). The mice received 5 mg/kg of TRi-1 or vehicle dissolved in PBS containing 2% DMSO every 2 d for 12 weeks. The tumor volume was calculated as tumor volume (cm^3^) = 0.5 × (length × width^2^). Humane endpoints were defined a priori; the mice were euthanized when the tumor burden exceeded 3.0 cm and/or when reaching other IACUC-defined criteria. When the mice were euthanized, the tumor burden was >3.0 cm.^3^ The overall survival rate was assessed from the date of tumor engraftment to the time of last observation or death. Euthanasia was performed by carbon dioxide (CO₂) inhalation using a gradual-fill method (displacement rate: 20%–30% of the chamber volume per minute) until respiratory arrest occurred, followed by a secondary physical method to ensure death. Death was confirmed by the absence of respiration and a heartbeat. All procedures were carried out in accordance with applicable veterinary guidelines, including the American Veterinary Medical Association (AVMA) Guidelines for the Euthanasia of Animals. All animal care and experimental procedures were conducted in accordance with the ethical guidelines approved by the Institutional Animal Care and Use Committee (IACUC) of Taipei Medical University (approval number: LAC-2018-0391).

### TGF-β1 ELISA

The supernatant from human H1299 and H1975 cells was collected 48 h after transfection with 10 μg of plasmids encoding TXNRD1 or the specified sgTXNRD1 knockdown in A549 cells. The concentration of the TGF-β1 ligand was measured using the Human TGF-β1 DuoSet ELISA kit (R&D Systems, DY240, USA) following the manufacturer's protocols.^47^

### Human growth factor antibody array

Human growth factor profiling was performed using a Human Growth Factor Antibody Array C1 kit (RayBiotech, AAH-GF-1-2, USA) according to the manufacturer’s instructions. This membrane-based, sandwich antibody array enables semi-quantitative detection of 41 human growth factors using chemiluminescence. Briefly, array membranes were equilibrated to room temperature and blocked with blocking buffer for 30 min. The diluted or undiluted samples were then incubated with the membranes under gentle rocking for 1.5–5 h at room temperature or overnight at 4 °C. After washing with the supplied wash buffers, the membranes were incubated with the biotinylated detection antibody cocktail for 1.5–2 h at room temperature or overnight at 4 °C, followed by incubation with HRP-conjugated streptavidin for 2 h at room temperature or overnight at 4 °C. Chemiluminescent signals were developed using Detection Buffers C and D and captured using a chemiluminescence imaging system.

### Statistical analysis

The experiments were performed with at least 3 independent biological replicates and 5 technical replicates each. For in vivo studies, each mouse was considered one biological replicate, and group sizes were *n* = 5 per group unless otherwise stated. These results were expressed as mean ± standard deviation. Statistical analyses were conducted using GraphPad Prism 8.0 statistical software (http://www.graphpad.com/scientific-software/prism). Statistical significance was set at *p < 0.05 and **p < 0.01, using a two-tailed Student’s *t*-test and a one-way analysis of variance (ANOVA). Kaplan‒Meier analysis was performed to evaluate the role of TXNRD1 in terms of survival outcomes.

## Supplementary Material

Supplementary Material Suppmentary_Figure_1.tif

Supplementary Material Supplementary_Table_1.docx

Supplementary Material Supplementary_Table_2.docx

Supplementary Material Author_Checklist.pdf

Supplementary Material Supplementary Material for review.docx

## Data Availability

This published article and its supplemental information files include all the data generated or analyzed during this study. The data from the Gene Expression Omnibus (GEO) database analyzed for this study is GSE316125. The dataset will be released upon publication. The raw data in this study may be obtained from the corresponding author upon reasonable request.
