# Supplementary material for: Thioredoxin reductase-1 facilitates cellular plasticity and stemness mediated by TGF-β1 in non-small cell lung cancer
Source: Cancer Biol Ther. 2026 Sep 8;27(1):2724630. doi: 10.1080/15384047.2026.2724630 (PMC13556941; doi:10.1080/15384047.2026.2724630)
Supplement: Supplementary file 2 [file KCBT_A_2724630_SM2296.docx]

**Cell Culture Reagents**

| **Reagent** | **Amount Used** | **Supplier** | **Catalogue No.** |
| --- | --- | --- | --- |
| Dulbecco's Modified Eagle Medium (DMEM) | Standard culture volume | Gibco-BRL, USA | 11965092 |
| RPMI 1640 | Standard culture volume | Corning, USA | 10-040-CV |
| Fetal Bovine Serum (FBS) | 10% (v/v) | Gibco-BRL, USA | 10270106 |
| Penicillin–Streptomycin | 1% (v/v) | Gibco-BRL, USA | 15140122 |
| L-Glutamine | 2 mM | Gibco-BRL, USA | 25030081 |
| Plasmocin® Mycoplasma Elimination Reagent | 25 μg/mL (treatment); 2.5 μg/mL (prophylactic) | InvivoGen, USA | ant-mpp |
| Puromycin | 2 μg/mL | InvivoGen, USA | ant-pr-1 |
| Blasticidin | 10 μg/mL | InvivoGen, USA | ant-bl-1 |
| Matrigel Basement Membrane Matrix | 1:1 (v/v) with cells | BD Biosciences, USA | 354234 |
| Polybrene | 8 μg/mL | Sigma-Aldrich, USA | H9268 |
| Neomycin (G418) | Standard selection concentration | Sigma-Aldrich, USA | A1720 |

**CRISPR/Cas9 and Transfection Reagents**

| **Reagent** | **Amount Used** | **Supplier** | **Catalogue No.** |
| --- | --- | --- | --- |
| Alt-R S.p. Cas9-GFP V3 Nuclease | 300 nM (final in RNP complex) | IDT, USA | 10007924 |
| Lipofectamine™ CRISPRMAX | Per manufacturer protocol | Invitrogen (Thermo Fisher Scientific), USA | CMAX00003 |
| jetPRIME® Transfection Reagent | Per manufacturer protocol | Polyplus-transfection, France | 101000046 |
| TXNRD1 Human Pre-designed siRNA Set A | 50 nM (final concentration) | MedChemExpress, USA | HY-RS15298 |
| Non-targeting Control siRNA | 50 nM (final concentration) | MedChemExpress, USA | HY-RS15298 (NC control included in set) |

**Western Blotting Reagents**

| **Reagent** | **Amount Used** | **Supplier** | **Catalogue No.** |
| --- | --- | --- | --- |
| RIPA Lysis Buffer | Standard volume per pellet | Sigma-Aldrich, USA | R0278 |
| Protease Inhibitor Cocktail | 1× | Roche, Switzerland | 11836153001 |
| Phosphatase Inhibitor | 1× | Roche, Switzerland | 04906845001 |
| Bradford Protein Assay Kit | Per manufacturer protocol | Bio-Rad, USA | 5000006 |
| PVDF Membrane | — | Millipore (Merck), USA | IPVH00010 |
| HRP-conjugated Goat anti-Rabbit IgG | 1:5000 | Thermo Fisher Scientific, USA | 31460 |
| HRP-conjugated Goat anti-Mouse IgG | 1:5000 | Thermo Fisher Scientific, USA | 31430 |

**Primary Antibodies (Western Blotting / IHC)**

| **Target** | **Dilution** | **Supplier** | **Catalogue No.** |
| --- | --- | --- | --- |
| TXNRD1 | 1:1000 (WB); 1:100 (IHC) | ABclonal (WB); Abcam (IHC) | A4725 (WB); EPNCIR129 (IHC) |
| Cas9 | 1:1000 | ABclonal, China | A23005 |
| E-Cadherin (24E10) | 1:1000 | Cell Signaling Technology, USA | 3195 |
| N-Cadherin (D4R1H) | 1:1000 | Cell Signaling Technology, USA | 13116 |
| Vimentin (D21H3) | 1:1000 | Cell Signaling Technology, USA | 5741 |
| Snail (C15D3) | 1:1000 | Cell Signaling Technology, USA | 3879 |
| Slug (C19G7) | 1:1000 | Cell Signaling Technology, USA | 9585 |
| Nanog (D73G4) | 1:1000 | Cell Signaling Technology, USA | 4903 |
| Sox2 (D6D9) | 1:1000 | Cell Signaling Technology, USA | 3728 |
| Oct-4 (D7O5Z) | 1:1000 | Cell Signaling Technology, USA | 83932 |
| Caspase-3 (D3R6Y) | 1:1000 | Cell Signaling Technology, USA | 14220 |
| Cleaved Caspase-3 (Asp175) | 1:1000 | Cell Signaling Technology, USA | 9661 |
| PARP (46D11) | 1:1000 | Cell Signaling Technology, USA | 9532 |
| Cleaved PARP (Asp214) | 1:1000 | Cell Signaling Technology, USA | 5625 |
| SMAD2/3 Antibody Sampler Kit | 1:1000 | Cell Signaling Technology, USA | 12747 |
| p-SMAD2/3 | 1:1000 | Cell Signaling Technology, USA | Included in #12747 |
| β-actin | 1:10000 | Sigma-Aldrich, USA | A5441 |

**RNA Extraction, RT-qPCR, and RNA-seq Reagents**

| **Reagent** | **Amount Used** | **Supplier** | **Catalogue No.** |
| --- | --- | --- | --- |
| TRIzol Reagent | 1 mL per 10 cm² culture | Thermo Fisher Scientific, USA | 15596026 |
| NucleoSpin RNA Kit | Per manufacturer protocol | Macherey-Nagel (MN), Germany | 740955.50 |
| First-Strand cDNA Synthesis Kit | Per manufacturer protocol | Thermo Fisher Scientific, USA | K1612 |
| SYBR Green PCR Master Mix | Per manufacturer protocol | Thermo Fisher Scientific, USA | 4309155 |
| Agilent 2100 Bioanalyzer RNA Kit | Per manufacturer protocol | Agilent Technologies, USA | 5067-1511 |

**Cytokine Detection and Growth Factor Array Reagents**

| **Reagent** | **Amount Used** | **Supplier** | **Catalogue No.** |
| --- | --- | --- | --- |
| Human TGF-β1 DuoSet ELISA Kit | Per manufacturer protocol | R&D Systems, USA | DY240 |
| Human Growth Factor Array (41-plex) | Per manufacturer protocol | RayBiotech, USA | AAH-GF-1-2 |

**In Vivo and Pharmacological Reagents**

| **Reagent** | **Amount Used** | **Supplier** | **Catalogue No.** |
| --- | --- | --- | --- |
| TXNRD1 Inhibitor TRi-1 | 5 mg/kg body weight, every 2 days | MedChemExpress, USA | HY-125006 |
| Isoflurane | Induction: 3%; Maintenance: 1.5% in O₂ | — | — |
| Dual-Glo® Luciferase Assay System | Per manufacturer protocol | Promega, USA | E2940 |
| B-27 Supplement | 1× | Gibco, USA | 17504044 |
| Recombinant Human bFGF | 20 ng/mL | Elabscience, China | ELABS-R-0011 |
| Recombinant Human EGF | 20 ng/mL | Elabscience, China | ELABS-R-0005 |
| SuperPicture Polymer Detection Kit | Per manufacturer protocol | Invitrogen, USA | 87965 |
