# Supplementary material for: Thioredoxin reductase-1 facilitates cellular plasticity and stemness mediated by TGF-β1 in non-small cell lung cancer
Source: Cancer Biol Ther. 2026 Sep 8;27(1):2724630. doi: 10.1080/15384047.2026.2724630 (PMC13556941; doi:10.1080/15384047.2026.2724630)
Supplement: Supplementary file 3 [file KCBT_A_2724630_SM2542.docx]

| **Supplementary Table 2** |  |  |  |
| --- | --- | --- | --- |
| **Factor** | **Adjusted HR** | **95% CI** | **p-value** |
| **TCGA_result (N=482)** |  |  |  |
| **TXNRD1 (log2)** | 1.102 | 1.018–1.192 | 0.016 |
| **Age** | 1.065 | 0.936–1.211 | 0.338 |
| **Sex (Male vs Female (ref))** | 0.92 | 0.721–1.174 | 0.502 |
| **Stage II vs Stage I (ref)** | 1.582 | 1.200–2.086 | 0.001 |
| **Stage III vs Stage I (ref)** | 2.32 | 1.662–3.239 | <0.001 |
| **Stage IV vs Stage I (ref)** | 2.255 | 1.460–3.482 | <0.001 |
| **Unknown vs Stage I (ref)** | 0.785 | 0.267–2.309 | 0.659 |
| **Pooled_microarray_Result_(with_batch_effect_adjustment) (N=801)** |  |  |  |
| **TXNRD1 (log2)** | 1.12 | 1.046–1.199 | 0.001 |
| **Age** | 1.26 | 1.162–1.365 | <0.001 |
| **Sex (Male vs Female (ref))** | 1.254 | 1.065–1.478 | 0.007 |
| **Stage II vs Stage I (ref)** | 1.439 | 1.212–1.709 | <0.001 |
| **Stage III vs Stage I (ref)** | 2.229 | 1.573–3.159 | <0.001 |
| **Stage IV vs Stage I (ref)** | 2.425 | 1.518–3.873 | <0.001 |
| **GSE68495 (N=442)** |  |  |  |
| **TXNRD1 (log2)** | 1.139 | 1.042–1.245 | 0.004 |
| **Age** | 1.246 | 1.120–1.386 | <0.001 |
| **Sex (Male vs Female (ref))** | 1.188 | 0.952–1.483 | 0.128 |
| **Stage II vs Stage I (ref)** | 1.261 | 1.010–1.573 | 0.04 |
| **Stage III vs Stage I (ref)** | 2.427 | 1.506–3.910 | <0.001 |
| **Stage IV vs Stage I (ref)** | 2.842 | 1.816–4.448 | <0.001 |
| **GSE50081 (N=128)** |  |  |  |
| **TXNRD1 (log2)** | 1.234 | 0.954–1.598 | 0.11 |
| **Age** | 1.167 | 0.943–1.444 | 0.155 |
| **Sex (Male vs Female (ref))** | 1.29 | 0.837–1.989 | 0.249 |
| **Stage II vs Stage I (ref)** | 2.104 | 1.343–3.297 | 0.001 |
| **GSE37745 (N=107)** |  |  |  |
| **TXNRD1 (log2)** | 1.057 | 0.863–1.295 | 0.592 |
| **Age** | 1.276 | 1.021–1.594 | 0.032 |
| **Sex (Male vs Female (ref))** | 1.266 | 0.856–1.870 | 0.237 |
| **Stage II vs Stage I (ref)** | 1.413 | 0.815–2.450 | 0.219 |
| **Stage III vs Stage I (ref)** | 1.833 | 1.098–3.060 | 0.02 |
| **Stage IV vs Stage I (ref)** | 1.329 | 0.482–3.664 | 0.583 |
| **GSE30219 (N=86)** |  |  |  |
| **TXNRD1 (log2)** | 1.073 | 0.845–1.364 | 0.563 |
| **Age** | 1.229 | 0.925–1.633 | 0.155 |
| **Sex (Male vs Female (ref))** | 1.01 | 0.541–1.887 | 0.974 |
| **Stage II vs Stage I (ref)** | 1.943 | 1.028–3.673 | 0.041 |
| **Stage III vs Stage I (ref)** | 1.106 | 0.203–6.033 | 0.907 |
| **GSE14814 (N=42)** |  |  |  |
| **TXNRD1 (log2)** | 1.108 | 0.864–1.420 | 0.418 |
| **Age** | 1.796 | 1.255–2.570 | 0.001 |
| **Sex (Male vs Female (ref))** | 2.725 | 1.277–5.817 | 0.01 |
| **Stage II vs Stage I (ref)** | 2.258 | 1.168–4.365 | 0.015 |

**Supplementary Table 2.** **Adjusted hazard ratios from multivariable Cox proportional hazards models for overall survival across TCGA and microarray cohorts.** Multivariable Cox regression was performed to assess the association of TXNRD1 expression with overall survival in the TCGA cohort (N = 482), the pooled microarray cohort with batch-effect adjustment (N = 801), and each GEO microarray dataset (GSE68495, GSE50081, GSE37745, GSE30219, and GSE14814; sample sizes as indicated). TXNRD1 expression was modeled as a continuous variable on the log2 scale. All models were adjusted for age, sex (male vs female; female as the reference), and pathological stage (Stage II–IV and/or Unknown, with Stage I as the reference where applicable). Results are presented as adjusted hazard ratios (HRs) with 95% confidence intervals (CIs) and corresponding p-values. Batch-effect adjustment was applied to the pooled microarray cohort before model fitting.
