# Supplementary material for: Thioredoxin reductase-1 facilitates cellular plasticity and stemness mediated by TGF-β1 in non-small cell lung cancer
Source: Cancer Biol Ther. 2026 Sep 8;27(1):2724630. doi: 10.1080/15384047.2026.2724630 (PMC13556941; doi:10.1080/15384047.2026.2724630)
Supplement: Supplementary file 4 [file KCBT_A_2724630_SM2546.docx]

| **Supplementary Table 1** | |
| --- | --- |
| **Real-Time PCR** |  |
| **Snail** | **CTTCCAGCAGCCCTACGAC** |
|  | **CGGTGGGGT TGAGGATCT** |
| **Slug** | **AGATGCATATTCGGACCCAC** |
|  | **CCTCATGTTTG TGCAGGAGA** |
| **Twist** | **AGCTACGCCTTCTCGGTCT** |
|  | **CCTTCTCTGG AAACAATGACATC** |
| **Nanog** | **CAACCAGACCCAGAACATCC** |
|  | **TTCCAA AGCAGCCTCCAAG** |
| **Oct4** | **ACCGAGTGAGAGGCAACC** |
|  | **TGAGAAAGGAGACCCAGCAG** |
| **Sox2** | **CGAGTGGAAACTTTTGTCGGA** |
|  | **TGTGCAGCGCTCGCAG** |
| **TGF-β1** | **CGTGGAGCTGTACCAGAAATA** |
|  | **TCCGGTGACATCAAAAGATAA** |
| **SMAD2** | **GGGTTTTGAAGCCGTCTATCAGC** |
|  | **CCAACCACTGTAGAGGTCCATTC** |
| **SMAD3** | **TGAGGCTGTCTACCAGTTGACC** |
|  | **GTGAGGACCTTGTCAAGCCACT** |
| **GAPDH** | **TGTTCGACAGTCAGCCGC** |
|  | **GGTGTCTGAGCGATGTGGC** |

**Supplementary Table 1.** **Primer sequences used for real-time quantitative PCR (RT–qPCR)**. The table lists the forward and reverse primer sequences used for RT–qPCR analysis of EMT- and stemness-related genes, including Snail, Slug, Twist, Nanog, Oct4, Sox2, TGF-β1, SMAD2, and SMAD3. GAPDH was used as the internal reference gene for normalization.
